# BALSAM—An Interactive Online Platform for Breath Analysis, Visualization and Classification

**DOI:** 10.3390/metabo10100393

**Published:** 2020-10-02

**Authors:** Philipp Weber, Josch Konstantin Pauling, Markus List, Jan Baumbach

**Affiliations:** 1 Department of Mathematics and Computer Science (IMADA), University of Southern Denmark, 5230 Odense M, Denmark; pweber@imada.sdu.dk; 2 LipiTUM Group, Chair of Experimental Bioinformatics, TUM School of Life Sciences Weihenstephan, Technical University of Munich, 85354 Freising, Germany; josch.pauling@wzw.tum.de; 3 Big Data in BioMedicine Group, Chair of Experimental Bioinformatics, TUM School of Life Sciences Weihenstephan, Technical University of Munich, 85354 Freising, Germany; markus.list@wzw.tum.de; 4 Chair of Experimental Bioinformatics, TUM School of Life Sciences Weihenstephan, Technical University of Munich, 85354 Freising, Germany

**Keywords:** breath analysis, machine learning, metabolite discovery, data-mining, metabolomics, biomarker, web-platform

## Abstract

The field of breath analysis lacks a fully automated analysis platform that enforces machine learning good practice and enables clinicians and clinical researchers to rapidly and reproducibly discover metabolite patterns in diseases. We present BALSAM—a comprehensive web-platform to simplify and automate this process, offering features for preprocessing, peak detection, feature extraction, visualization and pattern discovery. Our main focus is on data from multi-capillary-column ion-mobility-spectrometry. While not limited to breath data, BALSAM was developed to increase consistency and robustness in the data analysis process of breath samples, aiming to expand the array of low cost molecular diagnostics in clinics. Our platform is freely available as a web-service and in form of a publicly available docker container.

## 1. Introduction

Initially developed for defense applications in the US in the 1970s, ion-mobility-spectrometry is highly sensitive and used for the detection of explosives and drugs at airports [1]. Multi-capillary-column ion-mobility-spectrometry (MCC-IMS) couples two separation steps: A multi-capillary-column that separates the compounds by interaction with the column measured as retention time (RT) and further separation by the ion mobility spectrometer. The analytical mixture is pushed by a carrier gas through the MCC. Compounds with higher affinity for the stationary phase have a higher RT. In the ion mobility spectrometer, analytes are ionized and accelerated by an electric field until they reach the Faraday plate. Here, a reverse flow of drift gas slows down the ionized analytes [2]. The drift time is measured and forms the inverse reduced ion mobility (IRM)—i.e., the drift time in the spectrometer normalized for pressure, temperature, electric field strength and drift tube length [3,4]. By careful analysis of the spectra, one can infer the physical properties of the compound and quantify its concentration in the gas through its signal intensity. Frequently, the goal of odor and breath analysis is the identification of volatile organic compounds (VOCs) or the phenotype-association of VOC patterns in a binary case-control setting [5,6]. Such an associated VOC might not only reveal a biomarker for disease detection, but also highlight pathways and potential drug targets involved in the disease. Several technologies for VOC analysis have emerged in recent decades, allowing for potential non-invasive and rapid discovery of VOC patterns: electronic nose [7,8], gas chromatography-mass spectrometry (GC-MS) [9,10], liquid chromatography-mass spectrometry (LC-MS) [11,12] and multi-capillary-column ion-mobility-spectrometry [13,14,15] based devices are used for a majority of trials. MCC-IMS devices have seen an increase in popularity—e.g., in the detection of anesthetics in the workplace [16], accurate tracking and quantification of gaseous propofol concentration during surgery [17], identification of VOCs in idiopathic pulmonary fibrosis [18] and for breast cancer detection [19].

Other advantages of MCC-IMS are the relative robustness against moisture levels in exhaled air and their portability and compactness [20,21,22]. As compounds are not directly identifiable, MCC-IMS devices need a database or have to be applied jointly with other technology to label compounds. Notably, MCC-IMS devices can detect VOCs in concentrations of ppm to ppt range and can rapidly process breath samples within ten minutes per patient, allowing for rapid on-site analysis in hospitals [22]. They are also able to detect potentially confounding compounds from room air and surroundings [15]. Therefore, MCC-IMS devices have found widespread applications in medical research over the past two decades as an inexpensive and non-invasive technology [23].

In previous years, finding optimal combinations of pre-processing and analysis techniques that facilitate and reduce the need for manual interpretation of MCC-IMS data could not be fully solved [23,24,25]. While several techniques and tools have been established to facilitate these analyses, their use remains impractical for daily clinical practice for biomedical researchers who often lack the necessary time or are unfamiliar with a programming environment. Furthermore, results need to be robust and comparable, which require fully automated and standardized processing procedures. Thus, automatic analysis platforms have been developed for MCC-IMS data analysis, such as the IMS^2^ system [26]. Other tools, such as IMSDB [27] and CAROTTA [22], provide the identification of metabolites or unsupervised learning and correlation testing capabilities, but lack abilities for raw data handling and preprocessing. To our knowledge, there exist metabolomic analysis platforms for a variety of data types, but there are no actively maintained cloud-based analysis platforms for the processing and analysis of MCC-IMS data. The “Advanced Breath Analysis” platform ABA-Cloud enabled automatic and reproducible analyses of breath mass spectra [28]. It is no longer supported and hence only available as archived link at https://web.archive.org/web/20160321154103/http://aba.cloudminer.org/. While it provided a framework for the management and annotation of breath research studies, it also required expert knowledge about data structures and the programming environment. Therefore, it was infeasible to navigate for clinicians and clinical researchers despite the documentation. Beyond the analysis of MCC-IMS data, OpenMS [29,30] offers an extensive tool-set for data management and analysis of GC-MS and LC-MS studies through a modularized architecture. While providing a variety of methods to the community, it lacks support for MCC-IMS data. With BALSAM (Breath AnaLysis viSualizAtion Metabolite discovery), we present an accessible web-platform that integrates state-of-the-art preprocessing and analysis techniques (see [24,25,31]). BALSAM combines automatic cross-validation for reproducible machine learning, model performance estimation and an easily interpretable classification strategy to guide the biomedical decision-making process. Additionally, it does not require a background in computer science or statistics and greatly reduces the manual intervention required. We follow recommended techniques and provide capabilities for MCC-IMS preprocessing, peak detection and peak alignment [24,25,31]. Our main focus is the analysis of data from MCC-IMS devices. Furthermore, we offer feature selection and discovery in a fully automated fashion and provide an easily interpretable model in form of a decision tree which can be used to guide decision making and subsequent investigations. BALSAM is freely accessible at https://exbio.wzw.tum.de/balsam/.

## 2. Results

We present a new platform covering the data analysis workflow of MCC-IMS, ranging from raw data down to biomarker discovery and phenotyping. In the following section, we describe the possible analysis mode options and give two analysis examples for reference.

**Figure 1:**
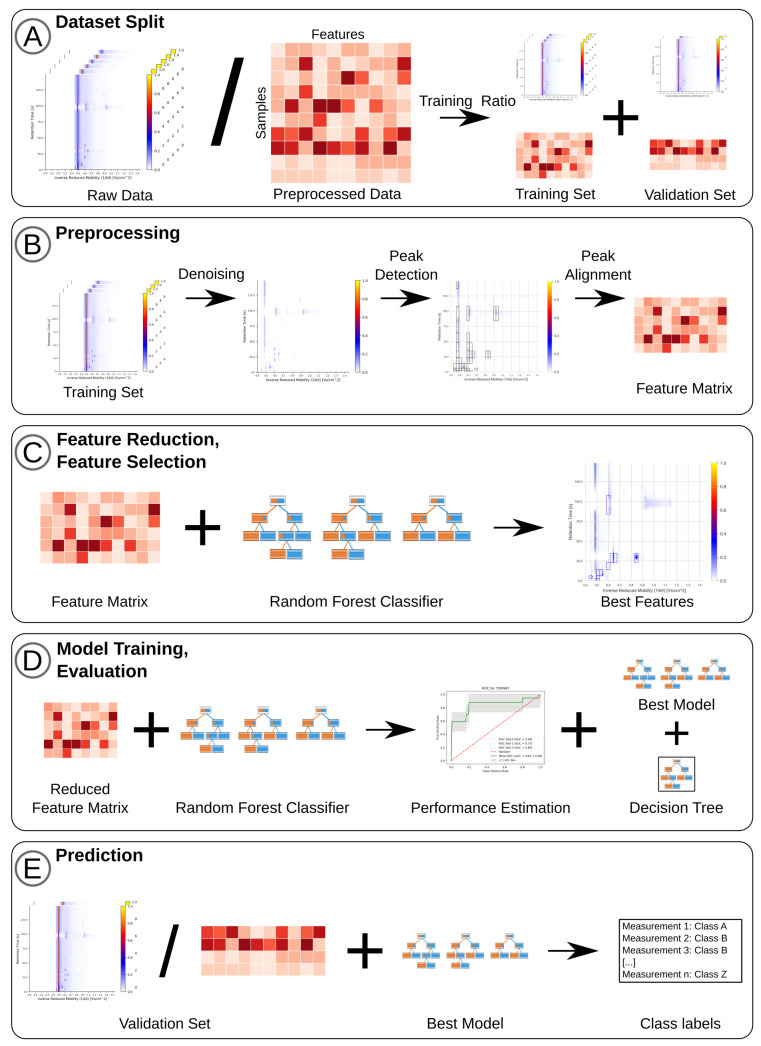
Full analysis workflow for measurements. (**A**) Dataset is uploaded and split into training and validation set according to selected training ratio. (**B**) Raw measurements are normalized and denoised. Peaks are detected and aligned to form a feature matrix. (**C**) The top n features are selected based on their informativeness and significance values across all patient classes. (**D**) Model performance is estimated in k-fold cross-validation, a prediction model is trained and a decision tree is built. (**E**) Previous preprocessing steps are applied to the validation set to extract the same features used in training and samples are classified.Full analysis workflow

Three analysis modes are available, each leading to a prediction model and allowing the users to download their results and plots (see Figure 1).

1. **Automatic**—Enables a fully autonomous analysis. Automatic selection of preprocessing and evaluation parameters. Selection of best-performing peak detection methods according to ROC-AUC (receiver operating characteristic area under curve) performance.
2. **Custom**—Offers guided and step-wise tuning of analysis parameters. Users can select between prediction models according to their requirements.
3. **Existing Results**—Allows usage of preprocessed data or previous analysis results and tuning of evaluation parameters. Feature matrices can be uploaded, skipping preprocessing and peak detection (see Supplementary Section S1.2 for file-format description).

### 2.1. Application Example: Candy Data-Set

**Figure 2:**
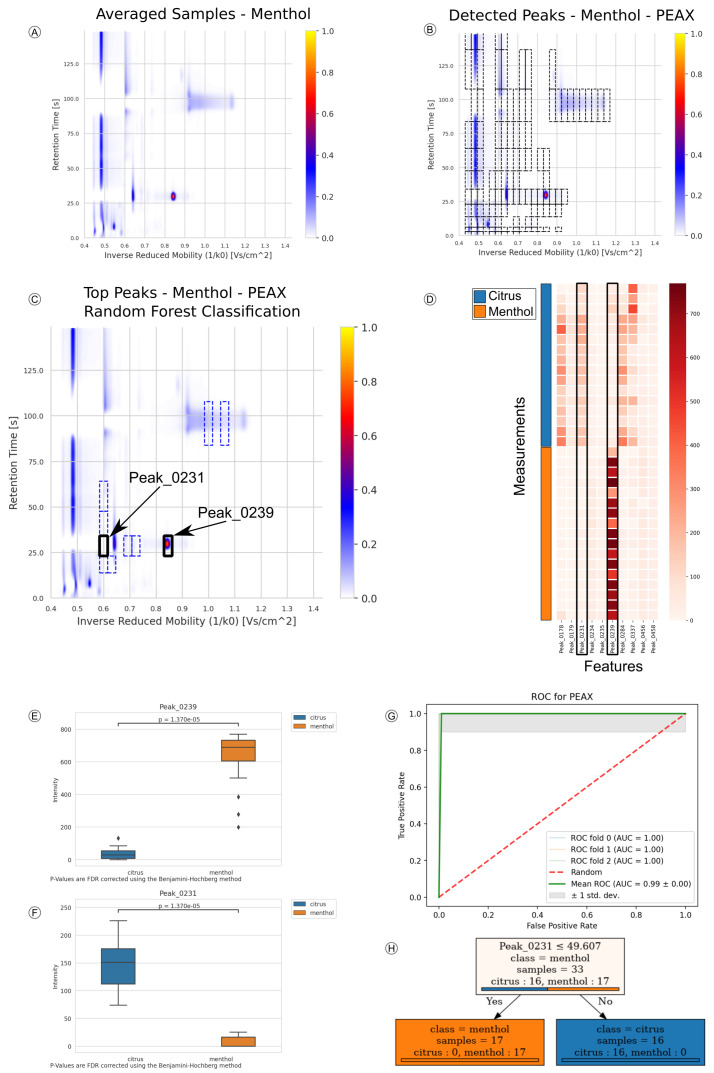
Stepwise results from processing the “Candy” dataset using PEAX as peak detection method. (**A**) Average normalized chromatogram for samples of class “menthol”. (**B**) Peaks detected by PEAX and aligned with Probe Clustering, overlay on average chromatogram of class “menthol”. (**C**) Top ten peaks ranked by random forest classifier. (**D**) Feature matrix of measurements used during cross-validation and model training. Columns of Peak_0231 and Peak_0239 are highlighted. (**E**,**F**) Boxplots of Peak_0231 and Peak_0239, intensities between “citrus” and “menthol” samples. (**G**) ROC curve during three-fold cross validation. The red line marks random performance, standard deviation is indicated. Average performance can be used to estimate actual model performance from training-set. (**H**) Decision tree incorporating the second highest ranked feature from random forest classification. Arrows guide the decision process by their label, coloring hints on the class composition of each node.

As an example, we apply the platform to the “Candy” dataset, where the dataset was split into a training:validation set with a 4:1 split (0.8 training ratio). We apply the “Automatic”-workflow with default parameters on the training set (see Supplementary Table S1 for a full list of parameters). After normalization and denoising, several reactant ion peak (RIP) artifacts stand out in the chromatogram (see Figure 2A). Among many potential peaks, these are also detected during peak detection using PEAX (see Figure 2B). According to the automatic selection of splits, three-fold cross-validation is selected and the top peaks are determined (here highlighted for “menthol” candies in Figure 2C). Subsequently, the default percentage threshold of 50% for feature reduction are applied to the training set, leading to a reduced feature matrix from which the top ten peaks (see Figure 2D) are reported and used for estimating the prediction-model performances.

*P*-values were computed with the Mann–Whitney U test and corrected using the Benjamini–Hochberg method for FDR with significance cutoff of 0.05. Peak_0239 (RT of 28.7, IRM of 0.846) shows elevated levels in “menthol” candies (q-value $1.4\times 10^{-5}$) (see Figure 2E).While the former peak is indicative of the “citrus” flavored candies, Peak_0231 (RT of 28.7, IRM of 0.6) shows a significant rise in intensity in “citrus” candies in comparison to the “menthol” class (q-value $1.4\times 10^{-5}$) (see Figure 2F). This dynamic can also be seen in Figure 2C,D, where the “citrus” Peak_0231 is missing in the menthol samples and reversely Peak_0239 is very intense. Subsequently, the top ten features for each peak detection-method were used to train the predictor, yielding PEAX as best prediction model with an average ROC-AUC of 0.99 ± 0.00 (see Figure 2G) and an accuracy of 0.97±0.03. The prediction of the measurements in the validation-set resulted in an entirely correctly labeled set. The created decision tree (Figure 2H) shows the process of classifying a breath sample into the two candy categories. “Citrus” Peak_0231 is at the top of the tree and perfectly distinguishes the measurements. The ten high-scoring peaks of interest are included in Supplementary Table S2 giving q-value and mean decrease in Gini-index for each peak as well as their coordinate definitions.

### 2.2. Application Example 2: COPD Data-Set

**Figure 3:**
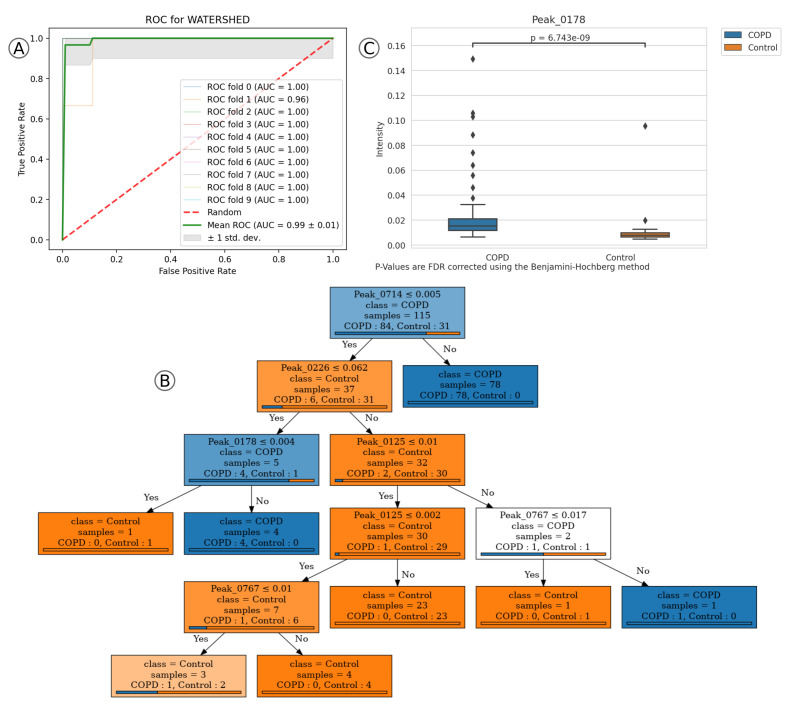
Receiver operating characteristic (ROC), boxplot and decision tree of top features of prediction model WATERSHED in “COPD” dataset. (**A**) ROC curve during the ten-fold cross validation. (**B**) Decision tree based on the highest ranked features from random forest classification. (**C**) Boxplot of Peak_0178 intensities.

Next, we apply the platform to the “COPD” dataset using a 9:1 ratio (0.9 training ratio) in training:validation split. Here we also use the “Automatic”-workflow with default parameters on the training set (see Supplementary Table S3 for a full list of parameters). Afterwards, we estimate the prediction-model performances on the training set using ten-fold cross-validation and the default percentage threshold of 50% for feature reduction. The model based on the WATERSHED method performs best and is automatically selected, reaching an average ROC-AUC of 0.99 ±0.01 (see Figure 3A) and an average accuracy of 0.96 ±0.04. *p*-values are again computed using the Mann–Whitney U test and corrected using the Benjamini–Hochberg method for FDR with significance cutoff of 0.05. Afterwards, we submit the validation set for prediction using the trained WATERSHED model and all samples are preprocessed. While the prediction model classifies all samples correctly into “COPD” and “Control”, the trained decision tree is not grown to its full possible depth and miss-classifies one “COPD” sample (see Figure 3B). It classifies the samples using five peaks: Peak_0714, Peak_0226, Peak_0178, Peak_0125 and Peak_0767 (see Supplementary Table S4 for the mean decrease in Gini index, q-values and full coordinate definitions). We compared the reported peaks with a previous study covering the same dataset from Hauschild et al. (2012) [32]. Of these five peaks, we could match Peak_0178 (RT of 18.5, IRM of 0.6) to Peak 103, which has been reported by both Hauschild et al. (2012) and in the original publication by Westhoff et al. (2011). In our trained model it has a mean decrease in Gini index of 0.039, a q-value of $6.7\times 10^{-9}$ and mostly lower intensity in “Control” cases (see Figure 3C), which indicates that additional analysis of the VOC is promising. Peak_0714 serves as root node in the decision tree, labels 78 of the 84 “COPD” samples and has a q-value of $7\times 10^{-10}$. As it shows higher intensities in “COPD” in comparison to “Control” samples, it should also be noted as a potential biomarker candidate. The decision tree subsequently classifies samples based on Peak_0226. If they have intensities larger than 0.062 they will likely be labeled as “Control”, with the exception of two “COPD” samples. It has a mean decrease in Gini index of 0.446 and a q-value of $1\times 10^{-7}$. The left branch of the decision tree distinguishes a single “Control” sample from the remaining four “COPD” samples by the intensity of Peak_0178. Westhoff et al. (2011) stated that most of their characteristic peaks in the dataset contain potential outliers or are outlier dominated. In comparison to them, we apply a stringent percentage threshold to the detected peaks, so our models are only trained on features which are conserved within at least 50% of samples of “Control” or “COPD” samples. While our prediction performance is good, we see an effect in the classification strategy of the trained decision tree. Many of the leaves contain only a single or a handful of samples. We can avoid this by tuning the decision tree training parameters, such as the number of samples required to split nodes or the minimum number of samples per leaf.

## 3. Discussion

Here we demonstrate two analysis examples of BALSAM. In the first application case with the “Candy” dataset, the two candy types were easily distinguishable by two very intense peaks clearly visible even in the raw spectra. Usually a very high performance of the classifier might be an indication that the model is overfitting. However, its performance on the held out validation-set was excellent, too. Therefore, we can be more confident in the learned features. While 25 samples per class is generally not enough for a comprehensive study, our analysis shows that the detected VOCs can serve as robust features for classification.

The second study highlights an application of the platform to a more realistic medical dataset. While we could show excellent classification performance, we also confirmed a previously suggested VOC among a set of candidates. Nonetheless, suggested VOCs require further identification and validation with a larger and more balanced sample set. Currently, there are almost three times as many “COPD” measurements in comparison to “Controls”, which could lead to a bias towards “COPD” measurements. Possible methods to balance the sample distribution without taking new measurements would be the application of an under-sampling technique for the majority class, which could discard potentially useful samples and information, or to use an oversampling technique for the minority class. While random oversampling is likely to increase overfitting methods, such as the Synthetic Minority Over-sampling Technique (SMOTE [33]), have been developed, which over-sample the minority class by generating new synthetic examples based on existing minority class instances. Future releases of BALSAM will include over-sampling techniques for imbalanced datasets. Using cross validation to estimate model performance will lead to a more accurate estimation of model performance in comparison to a simple train/test split. Decision trees are prone to overfitting, which can lead to poor classification performance on unseen data. One can avoid overfitting by tuning the decision tree’s parameters in a compromise, such as increasing the minimum number of samples required to split an internal node or increasing the number of samples required for leaf nodes. The resulting decision tree likely underfits the dataset, but may generalize better to unseen data. Tuning decision tree parameters will not affect the performance of the RFC, which is used for classification. Instead, the decision tree is used for interpretation of the model and, when it underfits the dataset, highlights the most robust features used for classification. Both RFC and the decision tree’s tendency to overfit can be mitigated by reducing the number of features used for training, so that they are significantly lower than the number of samples in the dataset. A way to detect overfitting is by validating the trained model on previously unseen data. Therefore, it is important to split the dataset into a training, test and validation set. As a consequence, we enforce a dataset split prior to using the analysis platform. After training the model, one can apply it in the prediction step to validate the performance on the data left out from training. Optimally, performance here is comparable to the estimation from the training step. If not satisfactory, training parameters need to be adjusted.

It is also important to be aware of possible biases in the study design, as these will be learned during training. A breath study can contain several confounding factors, such as age, sex or smoking status. BALSAM currently does not check for potential biases that could influence the classification performance. Additionally, if measurements are recorded using different devices, a scaling method should be applied to control for batch effects. Other hidden confounders, such as environmental conditions that differ between control and case groups if these were collected at different sampling sites or at different time points, can also influence the classification. In the future, we will investigate the use of blank samples of the environment to adjust for such confounders. When performance in training and prediction is satisfactory, exploration of the ranked peaks and training matrices can commence. Peaks with the highest mean decrease in Gini index and smallest q-values are the most discriminative features in the dataset and should be prioritized for follow-up analysis. While BALSAM does not contain a database for VOC identification, it is possible to follow up on the analysis with identification and monitoring of the associated compounds through the annotation of each peak with approximate coordinates in the chromatogram. While we analyzed and included several datasets in the platform, most of them are designed to easily separate with minimal VOCs. Thus, the platforms still lack validation with additional real world clinical data. The main focus of BALSAM is on MCC-IMS data, with rudimentary support for processing GC-MS and LC-MS raw data in the form of mzML and mzXML files. Therefore, we recommend using sophisticated software tailored for processing such data, such as OpenMS. That way, they can make use of other complementary peak alignment and imputation techniques and upload a feature matrix directly. These users can still benefit from BALSAM, as one can import a feature matrix for user-friendly machine learning. Hence, many spectrometry technologies can be supported and profit from the feature reduction and scoring methods. Additionally, users can generate visualizations to evaluate their sample sets and guide their search for potential biomarkers distinguishing phenotypes.

## 4. Materials and Methods

### 4.1. Testing and Validation

In addition to enabling the upload of user-owned datasets, we provide a small set of anonymized datasets as exemplary references. For each of them, we pre-split the dataset into a training and validation fraction. Similarly, users are able to split their uploaded datasets into training and validation sets, using our customized functions. Only datasets that were uploaded in this manner are possible to use, enforcing a clear separation between training and validation. Furthermore, we use cross validation on the training fraction as described in the methods section. Here, the training fraction of the datasets is further split into training and testing sections. User-provided datasets are temporarily stored on the server and automatically deleted after 30 days.

The “Candy” dataset is an artificially generated data set in a case/control setting using breath samples measured by an MCC-IMS machine (BreathDiscovery, B&S Analytik, Dortmund, Germany; SpiroScout with VOCan—v2.7; MCC of type OV5). Participants consumed one of two breath refreshment candies before their breath was sampled, one with a citrus-based flavor and the other with a menthol-based flavor. The samples contained 42 total samples, where 20 were from the “citrus” variety (HALLS, Honey Lemon Flavor, Mondelēz International) and 22 were from the “menthol” variety (HALLS, Ice Peppermint Flavor, Mondelēz International). Measurements were collected over a period of 3 years.

As a secondary evaluation dataset, we used the anonymous MCC-IMS dataset targeting the characterization of VOCs in chronic obstructive pulmonary disease (COPD) from Westhoff et al. [34]. Samples were recorded using an MCC-IMS device (BreathDiscovery, B&S Analytik, Dortmund, Germany; MCC of type OV5) in the Lung Hospital Hemer. COPD is a lung disease characterized by persistent breathing problems and reduced airflow due to airway and/or alveolar abnormalities. These are usually caused by long-term exposure to noxious particles or gases, such as tobacco smoke, fuel or air pollution [35]. The dataset consisted of 128 breath measurements, 93 from patients suffering from COPD, including patients with bronchial carcinoma (all labeled “COPD”) and 35 from a healthy control group (labeled “Control”). In addition to the “Candy” and the “COPD” datasets, we provide two more predefined sample sets for analysis (see Supplement Section S1.1).

### 4.2. Methods Overview

We illustrate the steps involved in the analysis of breath samples in Figure 1. All of these methods are available as user selection in the web-platform. After preprocessing and feature alignment, we support the import of precomputed results through feature matrices. Therefore, all feature reduction and feature selection methods (see Figure 1C,D) are available for samples from any origin that produces a feature matrix.

### 4.3. Preprocessing

Even during a controlled clinical trial, measurements are subject to many sources of technical variation. Investigators need to limit effects, such as instrumental noise, artifacts, room air conditions and mechanical drift [36]. Preprocessing can roughly be summarized into three parts:

1. Normalization and baseline correction;
2. De-noising and smoothing;
3. Peak detection.

In the following paragraphs, we will give an outline of available methods in this platform and give references to recommended literature explaining the techniques in more detail.

#### 4.3.1. Normalization and Baseline Correction

For preprocessing of raw MCC-IMS measurements we supply two methods: Intensity-normalization scales all spectra intensities to range [0,1] using the maximum intensity value in the RIP as the upper boundary. Baseline-correction/RIP detailing is used to remove the influence of the RIP. It reduces the effect of the RIP-tailing and lowers the baseline of the affected spectra by subtracting the 25% quantile intensity from all spectra (see [37] for more details).

#### 4.3.2. De-Noising and Smoothing

Noise-subtraction is applied to reduce technical noise and artifacts. Here, a fixed noise level is subtracted from all intensities. To determine this level we average the intensities with IRM values $<0.4\frac{Vs}{{cm}^{2}}$, which do not contain metabolite peaks and are un-affected by the RIP. Discrete-wavelet-transformation applies a compression algorithm to the spectra, decomposing the signals and applying a high and a low pass filter [38]. We make use of the Daubechies 8 wavelet and the implementation of PyWavelets [39].

The gaussian-filter removes noise by applying a fixed size Gaussian kernel and merges intensities with neighboring signals. Similarly, the median-filter removes noise by replacing intensities with the median of neighboring signals, and in case of the Savitzky–Golay-filter, replaces them with a weighted average [40]. The smoothing and de-noising effects can be seen in Figure 1B, which shows an MCC-IMS measurement prior to application of de-noising steps and after.

#### 4.3.3. Peak Detection

After de-noising, we can extract the features in each measurement using techniques ranging from image and signal processing to methods specifically developed for MCC-IMS data. In Figure 1B the detected peaks in a preprocessed measurement are indicated in the third panel. PEAX [41] is a non-commercial automated peak extraction method for MCC-IMS measurements. Its core idea is to extract lower dimensional peak models from the spectra and to merge them into two-dimensional peak models. In the Tophat-method [42] peaks are extracted in a two-step process: Tophat-filtering and local maxima extraction. In the first step, a noise-threshold is applied that removes all intensities below this user-supplied threshold. Subsequently, a 2D-window is created that highlights areas of high intensities. In the second step, the local maximum of each overlapping window area is extracted and saved as intensity value. JIBB is a naive peak extraction approach implemented by us. It considers an area a peak if its intensity is 1.5 times above the mean noise level and a number of consecutive signals are raising continuously in the IRM and RT directions while reflecting the inverse behavior when moving away from the local maximum. The watershed approach mimics a falling water-level that is lowered from maximum intensity value until it reaches the noise level. Local maxima reaching out of the water-level are labeled as peaks until the noise threshold is reached. A similar implementation is used in the IPHEx (IMS Peaklist & Heatmap Explorer) software [43]. Using the VisualNow-layer-method, peaks are extracted in rectangles based on the positions provided in the layer/annotation file. This enables the import of the annotation file from analyses with the commercial Visual Now software (B&S Analytik, Dortmund, Germany) [44].

### 4.4. Peak Alignment

Peak alignment is the process of identifying identical peaks across measurements despite slight shifts in their position. This makes it possible to map these peaks to the same metabolite throughout an experiment. Two approaches, Probe-Clustering and DBSCAN (Density-Based Spatial Clustering of Applications with Noise) [45] are available in the platform (for a direct comparison see Figure S1). Probe-Clustering is closely related to the “Grid-Squares” approach termed by Horsch et al. [24] and makes use of a RT scaling method similar to the implementation in Visual Now [44]. Its application is vital—the peak is assigned to specific peaks and needs to be deterministic and should not differ between runs, as we rely on peaks as a proxy for metabolites during classification.

As illustrated in Figure S2A, we create a fixed grid with two parameters, a constant grid width *w* and a scaling factor $s_{rt}$ for RT scaling according to Equation (1). Both parameters are user supplied and used for all measurements in an experiment. Default values work well across a wide range of tested data sets. RT scaling is necessary, as compounds more strongly interacting with the columns are released over a longer period of time, leading to peaks with higher RT being spread out over a longer RT period, which we counteract by linearly scaling the grid height. Peaks are assigned to cells based on the position of the maximum intensity and labeled by that cell’s peak id. This assigns unique identifiers to the same peak positions between measurements, resulting in consistent identifiers for all measurements and between experiments. The standard grid can be seen superimposed on top of a spectrogram in Figure S2B. The grid has an upper RT limit of 2000 s and a maximum IRM of 1.6 $\frac{Vs}{cm^{2}}$. The height *h* of each row *q* is defined by $h_{q}=H\left(q+1\right)-H\left(q\right)\;\forall q\in N,q\geq 0$, where *H* is defined as:

(1) $$\begin{array}{cc} H(0) & =0.0s\\ H(1) & =3.0s\\ H(q) & =H\left(q-1\right)+\left(1+s_{rt}\right)*H\left(q-1\right) \end{array}$$

Peak ids are numbered from left to right and bottom to top. After peak detection, peak ids are assigned based on the position of maximum intensity in each peak and coordinates are set to the center of each cell.

In comparison to a static grid, the main component in the DBSCAN algorithm is the notion of core samples. In this context, we consider each detected peak in each measurement as a sample. Such a sample is a core sample, when it lies in an area of high density, which is true when there are *min_samples* samples within a distance of *eps*. Both *min_samples* and *eps* are user supplied parameters, but default values worked well during testing. Core samples form a cluster by recursively including all other samples within a distance of *eps*. All samples that are not included in clusters are considered noise [45]. We apply DBSCAN to the peak positions determined in the peak detection process. The mean position of formed clusters is noted and they are labeled based on the probe clustering grid with default parameters. Similarly, peaks which are considered noise by the DBSCAN algorithm, and are therefore not part of any cluster, are assigned a peak id based on the same grid.

### 4.5. Feature Reduction

**Figure 4:**
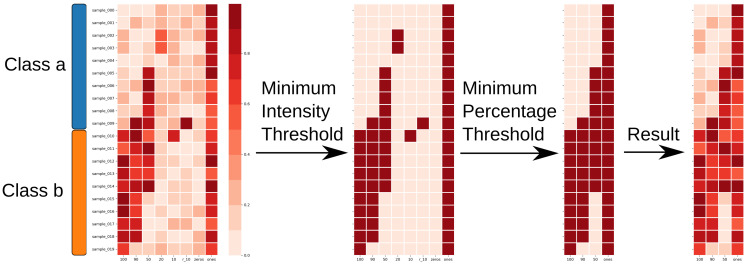
Feature reduction applied with minimum intensity threshold of 0.3 and minimum percentage threshold of 30% to a feature matrix with samples from two classes. Columns that do not fulfill both thresholds are removed from the feature matrix.

Often the number of samples in an analysis is smaller than the number of features present in the measurements. Such a mismatch can lead to overfitting of the model parameters to the training data and reduce performance in the test and validation data. Therefore, we apply feature reduction and only keep the most predictive features (see Figure 1C and Figure 4). For this, we consider the user defined class labels for each measurement. We remove features based on two thresholds: the minimum intensity threshold and the percentage threshold. The former is applied to remove noise, while the latter enforces that a feature needs to be present in at least percentage threshold percent of measurements of a given class label. When applying a percentage threshold of 30% on a set with class labels *a* and *b*, each feature needs to be present in at least 30% of either class *a* or class *b* to be included in the subsequent analysis. Applying more stringent percentage cutoffs leads to the inclusion of highly conserved and therefore more representative features for each class.

### 4.6. Performance Estimation

**Figure 5:**
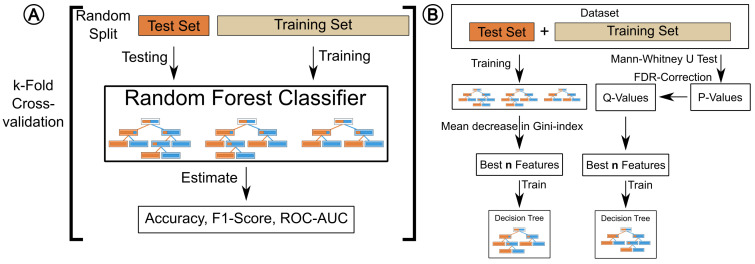
Illustration of performance estimation and feature scoring using cross-validation. (**A**) During k-fold cross-validation *k* Random forest classifiers (RFCs) are trained and their performances are calculated on the test set, estimating accuracy, F1-Score and ROC-AUC (receiver operating characteristic area under curve) (**B**) Feature scores are computed using the Mann–Whitney-U Test with FDR-correction and a RFC. The features are ranked, and the highest-scoring ones are used to train a decision tree classifier for each ranking method.Illustration of performance estimation and feature scoring

After feature reduction, the training portion of the dataset is further split into a training and test-set using *k*-fold cross-validation in a balanced fashion, keeping the approximate class distribution close to that of the full dataset (see Figure 5A). While *k* is user-supplied, it defaults to dynamically fit the sample set size during parameter selection, with a maximum value of ten. If less than ten samples are available in the minority class, cross validation is omitted. For larger datasets, the number of folds *k* is increased for every five samples with an upper bound of ten folds. The idea behind this is that the test-set will always have at least 5 samples per class label to evaluate performance. So, a dataset with 15 samples for class “a” and 20 samples for class “b” will be evaluated using three-fold cross validation. Random forest classifiers (RFC) are trained on the training set, and their performance is estimated using the test set of the split. In addition to specificity and sensitivity, we calculate the accuracy, F1-Score and receiver operating characteristic (ROC) to measure model performance. We average the classifier performance over the *k*-splits and generate plots for visualization.

### 4.7. Feature Selection

We offer two complimentary approaches for feature selection. The first method is based on a Mann–Whitney U test [46,47] with the aligned peak intensities as input. The resulting p-values are transformed into q-values using the Benjamini–Hochberg false discovery rate (FDR) method [48]. Subsequently, BALSAM trains a RFC on the full dataset and extracts the mean decrease in Gini index as feature importance measure (see Figure 5A). It will serve as classification model in the prediction step. For both methods, features are ranked and only the top *n* features are kept, where *n* is a user-defined number. For each feature selection method, a decision tree is trained using these top features as input. Their visual representation can be used as a guide for classifying samples, but also to approximate the decision-making process of the RFC. Afterwards, boxplots are generated and plots are formed to indicate the best features for each sample class.

### 4.8. Prediction

Using the RFC trained on the full dataset, the platform predicts the class labels of a new dataset. When applying the prediction to raw measurements, it will also perform the same preprocessing steps to reconstruct the same features as used during training of the prediction model. Using peak detection results, BALSAM will apply the same peak alignment method to get the identical feature positions, while using the given labels in case a feature matrix serves as input. This results in the assignment of class labels on a per measurement basis, presented in the final step of the analysis.

### 4.9. Metabolite Discovery

The discriminative features associated with phenotype and disease labels and the corresponding peak positions can serve as the basis for further metabolite discovery. The decision trees highlight the most discriminative features in the training set. Using a tool such as IMSDB [27] or by coupling analysis with additional gas-chromatography, unknown peaks in MCC-IMS can additionally be identified [49].

### 4.10. Implementation

BALSAM (v1.0) is implemented in the Python programming language (v3.6) [50] and makes extensive use of BreathPy (v0.8) [51], SciPy (v1.4) [52], scikit-learn (v0.22) [53] and PyOpenMS (v2.4) [54]. We further make use of Django (v2.2) [55], PostgreSQL (v9.5) [56], Celery (v3.1) [57], Jobtastic (v2.1) [58], NumPy (v1.18) [59], Pandas (v1.0) [60], Statsmodels (v0.11) [61], Matplotlib (v3.2) [62] and Seaborn (v0.10) [63].

### 4.11. Software Availability and License

BALSAM is available as web-service at https://exbio.wzw.tum.de/balsam/. Users that do not wish to upload their data can host BALSAM locally by deploying our docker container from https://hub.docker.com/repository/docker/philmaweb/balsam_docker. The source code for BALSAM is released under Gnu Public License version 3 at https://github.com/philmaweb/balsam_django and includes BreathPy. BreathPy contains binaries for PEAX, which is a free software for academic use only.

## 5. Conclusions

With BALSAM, we present a freely available software solution for the investigation of MCC-IMS data. It covers all steps of a typical analysis and integrates the latest preprocessing and analysis technologies for MCC-IMS data. Users can rapidly process samples from various spectrometry platforms through the existing results mode. The incorporation of automatic cross-validation, model performance estimation and validation leads to higher reproducibility. Furthermore, users are presented with an easily interpretable classification strategy to support decision-making processes. By eliminating manual intervention as much as possible, we promote rapid scientific analysis and reproducibility to researchers using a variety of sampling techniques. Therefore, BALSAM fulfills initial motivation criteria and is an important step to bring breath analysis into clinics as a robust, low-cost diagnostic tool capable of supporting traditional diagnostics, especially for respiratory conditions.

## Supplementary Materials

The supplementary materials are available at https://www.mdpi.com/2218-1989/10/10/393/s1.

## Abbreviations

The following abbreviations are used in this manuscript:

BALSAM	Breath AnaLysis viSualizAtion Metabolite discovery
COPD	Chronic Obstructive Pulmonary Disease
DBSCAN	Density-Based Spatial Clustering of Applications with Noise
FDR	False Discovery Rate
GC-MS	Gas Chromatography-Mass Spectrometry
IMS	Ion-Mobility-Spectrometry
IRM	Inverse Reduced ion Mobility
LC-MS	Liquid Chromatography-Mass Spectrometry
MCC	Multi-Capillary-Column
RFC	Random Forest Classifier
RIP	Reactant Ion Peak
ROC-AUC	Receiver Operating Characteristic Area Under Curve
RT	Retention Time
SMOTE	Synthetic Minority Over-sampling Technique
VOC	Volatile Organic Compound
