# Supplementary material for: BALSAM—An Interactive Online Platform for Breath Analysis, Visualization and Classification
Source: Metabolites. 2020 Oct 2;10(10):393. doi: 10.3390/metabo10100393 (PMC7601018; doi:10.3390/metabo10100393)
Supplement: Supplementary file 1 [file metabolites-10-00393-s001.zip › supplements_balsam_review01.pdf]

1. Supplementary Material

1.1. Supplementary Datasets

We provide a small set of anonymized datasets as analysis examples. Each of them is split into a random training and validation fraction, allowing for additional model evaluation after training a prediction model from extracted features.
The **mouthwash** dataset is available in the form of an anonymized feature matrix. It contains 49 measurements, where each belongs to one of seven classes. Classes are assigned by the brand of mouthwash used, where a series of seven breath measurements are taken after controlled periods following application of the solution. The **algae** dataset is composed of 19 samples of single cell MS experiments from *Scrippsiella trochoidea* and available as featureXML files. The algae were raised in 4 different conditions: light, dark, nitrogen-limited and replete (post nitrogen-limited), in which the authors could identify significant differences in metabolome and lipid complements [1].

1.2. Supported data formats

The BALSAM platform supports several data formats as inputs. To ease the transfer of data we enforce the upload of zip-archives without any subdirectories. Each zip-archive requires a class label file and data in form of raw measurements or a feature matrix. Their definitions are outlined below and in the documentation section of the website.

MCC-IMS Measurements
Each MCC-IMS measurement is stored in a custom comma-separated values (CSV) file format. The first part of the file is a header holding information about the sample, sampling procedure, device specifications and gas flows. The later part of the format holds the intensity values in a matrix, where columns hold the label for inverse reduced ion-mobility and rows are labeling the retention time. For a complete reference to the specific file format see Baumbach *et. al* (2001) [2] and Vautz *et. al* (2008) [3]. Files in the archive are expected to end with the suffix *"_ims.csv"*.

MZML and MZXML Measurements
Raw files from GC-MS or LC-MS raw measurements can be imported using the mzML and mzXML file formats. Vendor-specific formats can be converted to the open formats using the freely accessible tool *ProteoWizard* [4] or the R-library *mzR*. Expected suffixes are *".mzML"* and *".mzXML"*.

Class Label File
The class label file is required for the supervised part of the analysis, namely the feature extraction and reduction, as well as the creation of prediction models and the estimation of the model performance. We support three data formats for parsing a class label file

and scan the archive for a file with the suffixes *"class_labels.csv"*, *"class_labels.tsv"* or *"class_labels.txt"*. The first row should be a header row in the class labels file, the first column should reference all measurement names in the zip-archive, while the second column assigns the class to each measurement. CSV-files should use commas as separation symbol, TSV-files should use tabs as separation and TXT-files should use a single whitespace as separation. We provide example files in the documentation section of the platform.

Peak Layer File

A peak-layer-file defines the peak positions for extraction of intensities and is used as basis for the VisualNow-layer peak detection method. When contained in the dataset, it needs to end with the suffixes *"_layer.csv"* or *"_layer.xls"*, where a CSV-file uses commas as separation symbol between columns and the XLS-file is the proprietary ms-office format. Two column schemes are supported and documented in the documentation section of the platform.

Feature Matrix

A feature matrix is the result of a successful peak-detection and alignment step, listing intensities for peak ids in each measurement. The first line holds the header, rows indicate the associated measurement while the columns define the peak ids. Each feature matrix file should end with the suffix *"_feature_matrix.csv"*. If the name contains any of the peak detection result names TOPHAT, PEAX, WATERSHED, JIBB or VISUALNOWLAYER, it will be assigned as peak detection method in favor of CUSTOM.

1.3. Peak Alignment Plots

Figure S1 highlights the differences between the two available peak alignment methods. Identical measurements were used and the same peak layer file served as peak detection method, while the alignment methods were varied for the plots in Figure S1. While the standard grid is clearly visible in the probe clustering Figures S1 A and B, Figures S1 C and D show smaller and partly overlapping peak definitions. Furthermore, the DBSCAN based method does not scale the retention time linearly. Instead it relies on the distances between the peak centers and assigns a minimum cluster height of 6.0s, which leads to the depicted uniform cluster heights.

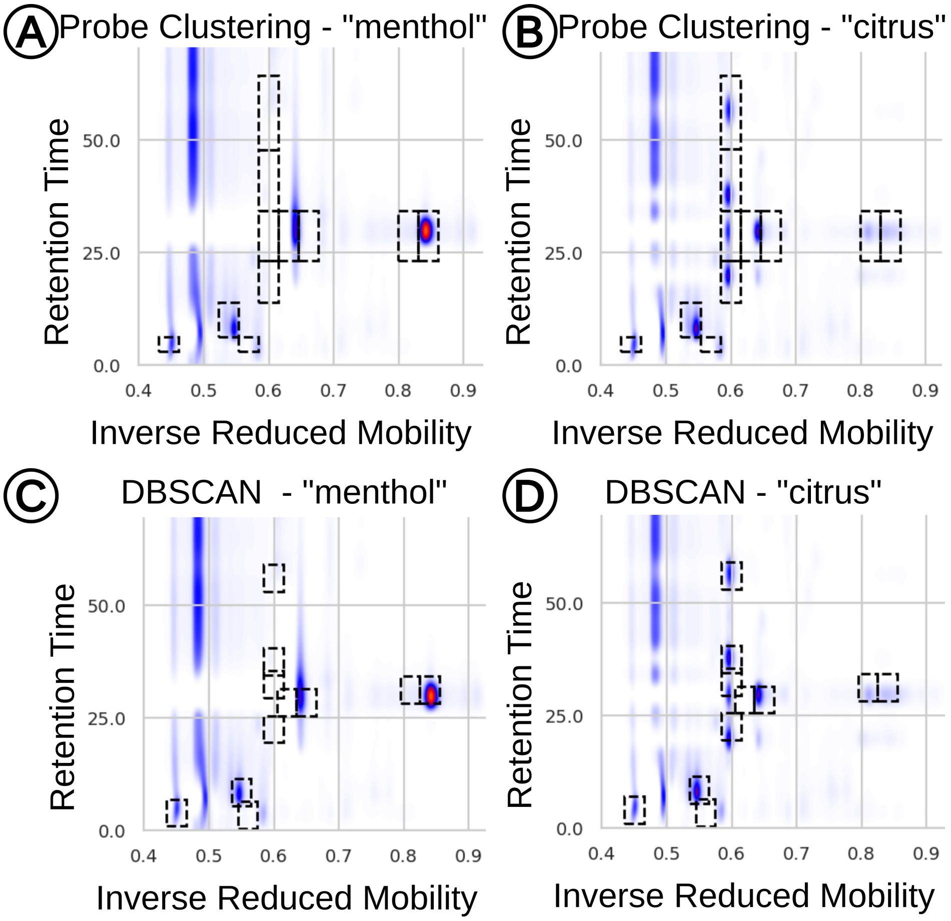

Figure S1: Comparison of peak definitions from probe clustering and DBSCAN on top of chromatograms (A) Peak ids based on probe clustering for "menthol" candies (B) Peak ids based on probe clustering for "citrus" candies (C) Peak ids based on DBSCAN clustering for "menthol" candies (D) Peak ids based on DBSCAN for "citrus" candies.

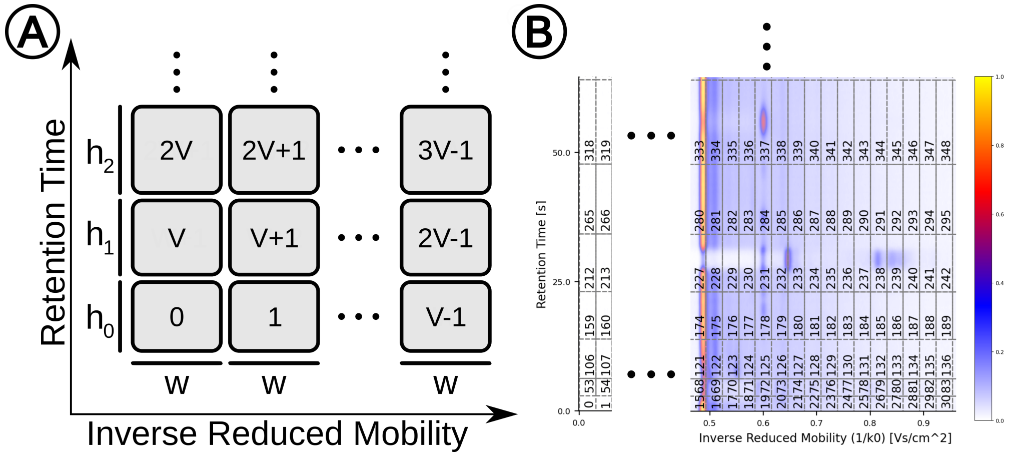

Figure S2: Peak id definition of probe clustering method. Peak ids are displayed in each cell. (A) Determination principle of probe clustering peak ids using cell height h and width w, where V cells are contained in each row (B) Probe clustering grid with standard parameters on top of a chromatogram.

Figure S2 shows the definition of peak ids based on the probe clustering method. In Figure S2 A the principle of peak id determination is shown and applied in Figure S2 B.

1.4. Candy dataset supplements

Table S1: Configuration parameters for candy analysis run. No parameters passed is indicated by {}.

Method	Parameters
JIBB	{'noise_threshold': 1.5, 'range_ivr': 5, 'range_rt': 7}
PEAX	{}
TOPHAT	{'noise_threshold': 1.4}
WATERSHED	{'noise_threshold': 1.5}
PROBE_CLUSTERING	{'threshold_inverse_reduced_mobility': 0.015, 'threshold_scaling_retention_time': 0.1}
MEDIAN_FILTER	{'kernel_size': 9}
GAUSSIAN_FILTER	{'sigma': 1}
SAVITZKY_GOLAY_FILTER	{'window_length': 9, 'poly_order': 2}
CROP_INVERSE_REDUCED_MOBILITY	{'cutoff_1ko_axis': 0.4}
DISCRETE_WAVELET_TRANSFORMATION	{'level_inverse_reduced_mobility': 4, 'level_retention_time': 2}
BASELINE_CORRECTION	{}
INTENSITY_NORMALIZATION	{}
FDR_CORRECTED_P_VALUE	{'n_of_features': 10, 'benjamini_hochberg_alpha': 0.05}
DECISION_TREE_TRAINING	{'max_depth': 5, 'min_samples_leaf': 1, 'min_samples_split': 2}
REMOVE_PERCENTAGE_FEATURES	{'noise_threshold': 0.0001, 'percentage_threshold': 0.5}
RANDOM_FOREST_CLASSIFICATION	{'n_of_features': 10, 'n_splits_cross_validation': 3, 'n_estimators_random_forest': 2000}

Table S2: Top 10 features in the candy example ranked by q-value and mean gini decrease. IRM and RT give center coordinates for each peak id. Bold peak ids indicate usage in decision tree. RFC: random forest classifier, IRM: inverse reduced ion mobility, RT: retention time.

Evaluation Method	Peak Detection Method	Class Comparison	Peak Id	Gini Decrease	q-value	IRM	Radius IRM	RT	Radius RT
RFC	PEAX	overall	Peak_0239	0.139	1.3700707E-05	0.846	0.015	28.7	5.6
RFC	PEAX	overall	**Peak_0231**	0.126	1.3700707E-05	0.6	0.015	28.7	5.6
RFC	PEAX	overall	Peak_0284	0.087	1.5645528E-05	0.6	0.015	41.1	6.8
RFC	PEAX	overall	Peak_0337	0.073	1.5645528E-05	0.6	0.015	56	8.2
RFC	PEAX	overall	Peak_0178	0.066	1.5645528E-05	0.6	0.015	18.5	4.6
RFC	PEAX	overall	Peak_0179	0.06	1.5645528E-05	0.631	0.015	18.5	4.6
RFC	PEAX	overall	Peak_0235	0.06	0.000021864	0.723	0.015	28.7	5.6
RFC	PEAX	overall	Peak_0234	0.04	0.0002049209	0.692	0.015	28.7	5.6
RFC	PEAX	overall	Peak_0456	0.038	0.0006822718	1	0.015	95.9	12
RFC	PEAX	overall	Peak_0459	0.031	8.8390927E-05	1.092	0.015	95.9	12

Table S3: Configuration parameters for the COPD analysis run. No parameters passed is indicated by {}.

Method	Parameters
JIBB	{'noise_threshold': 1.5, 'range_ivr': 5, 'range_rt': 7}
PEAX	{}
TOPHAT	{'noise_threshold': 1.4}
WATERSHED	{'noise_threshold': 1.5}
PROBE_CLUSTERING	{'threshold_inverse_reduced_mobility': 0.015, 'threshold_scaling_retention_time': 0.1}
MEDIAN_FILTER	{'kernel_size': 9}
GAUSSIAN_FILTER	{'sigma': 1}
SAVITZKY_GOLAY_FILTER	{'window_length': 9, 'poly_order': 2}
CROP_INVERSE_REDUCED_MOBILITY	{'cutoff_1ko_axis': 0.4}
DISCRETE_WAVELET_TRANSFORMATION	{'level_inverse_reduced_mobility': 4, 'level_retention_time': 2}
BASELINE_CORRECTION	{}
INTENSITY_NORMALIZATION	{}
FDR_CORRECTED_P_VALUE	{'n_of_features': 10, 'benjamini_hochberg_alpha': 0.05}
DECISION_TREE_TRAINING	{'max_depth': 5, 'min_samples_leaf': 1, 'min_samples_split': 2}
REMOVE_PERCENTAGE_FEATURES	{'noise_threshold': 0.0001, 'percentage_threshold': 0.5}
RANDOM_FOREST_CLASSIFICATION	{'n_of_features': 10, 'n_splits_cross_validation': 10, 'n_estimators_random_forest': 2000}

Table S4: Top 10 features in the COPD example ranked by mean gini decrease. IRM and RT give center coordinates for each peak id. Bold peak ids indicate usage in decision tree. RFC: random forest classifier, IRM: inverse reduced ion mobility, RT: retention time.

Evaluation Method	Peak Detection Method	Class Comparison	Peak Id	Mean Gini Decrease	q-value	IRM	Radius IRM	RT	Radius RT
RFC	WATERSHED	overall	**Peak_0714**	0.165	7.11E-10	0.785	0.015	296.5	31
RFC	WATERSHED	overall	**Peak_0767**	0.104	7.00E-12	0.785	0.015	365.1	37.5
RFC	WATERSHED	overall	Peak_0664	0.069	5.41E-09	0.877	0.015	239.9	25.6
RFC	WATERSHED	overall	Peak_0717	0.049	0.1506412691	0.877	0.015	296.5	31
RFC	WATERSHED	overall	**Peak_0125**	0.037	6.72E-05	0.6	0.015	10.1	3.8
RFC	WATERSHED	overall	**Peak_0178**	0.033	6.74E-09	0.6	0.015	18.5	4.6
RFC	WATERSHED	overall	**Peak_0226**	0.032	1.09E-07	0.446	0.015	28.7	5.6
RFC	WATERSHED	overall	Peak_0389	0.032	1.09E-07	0.569	0.015	74	9.9
RFC	WATERSHED	overall	Peak_0288	0.026	5.18E-09	0.723	0.015	41.1	6.8
RFC	WATERSHED	overall	Peak_0179	0.025	1.18E-08	0.631	0.015	18.5	4.6

References

[1] Sun, M.; Yang, Z.; Wawrik, B. Metabolomic Fingerprints of Individual Algal Cells Using the Single-Probe Mass Spectrometry Technique. *Frontiers in Plant Science* **2018**, *9*, 571. doi:10.3389/fpls.2018.00571.

[2] Baumbach, J.I.; Davies, A.N.; Lampen, P.; Schmidt, H. JCAMP-DX. A standard format for the exchange of ion mobility spectrometry data (IUPAC

Recommendations 2001). *Pure and Applied Chemistry* **2001**, *73*, 1765–1782. doi:10.1351/pac200173111765.

[3] Vautz, W.; Bödeker, B.; Bader, S.; Baumbach, J.I. Recommendation of a standard format for data sets from GC/IMS with sensor-controlled sampling. *International Journal for Ion Mobility Spectrometry* **2008**, *11*, 71–76. doi:10.1007/s12127-008-0010-9.

[4] Chambers, M.C.; Maclean, B.; Burke, R.; Amodei, D.; Ruderman, D.L.; Neumann, S.; Gatto, L.; Fischer, B.; Pratt, B.; Egertson, J.; Hoff, K.; Kessner, D.; Tasman, N.; Shulman, N.; Frewen, B.; Baker, T.A.; Brusniak, M.Y.; Paulse, C.; Creasy, D.; Flashner, L.; Kani, K.; Moulding, C.; Seymour, S.L.; Nuwaysir, L.M.; Lefebvre, B.; Kuhlmann, F.; Roark, J.; Rainer, P.; Detlev, S.; Hemenway, T.; Huhmer, A.; Langridge, J.; Connolly, B.; Chadick, T.; Holly, K.; Eckels, J.; Deutsch, E.W.; Moritz, R.L.; Katz, J.E.; Agus, D.B.; MacCoss, M.; Tabb, D.L.; Mallick, P. A cross-platform toolkit for mass spectrometry and proteomics. *Nature Biotechnology* **2012**, *30*, 918–920. doi:10.1038/nbt.2377.
