# Supplementary figures and images for: BALSAM—An Interactive Online Platform for Breath Analysis, Visualization and Classification
Source: Metabolites. 2020 Oct 2;10(10):393. doi: 10.3390/metabo10100393 (PMC7601018; doi:10.3390/metabo10100393)

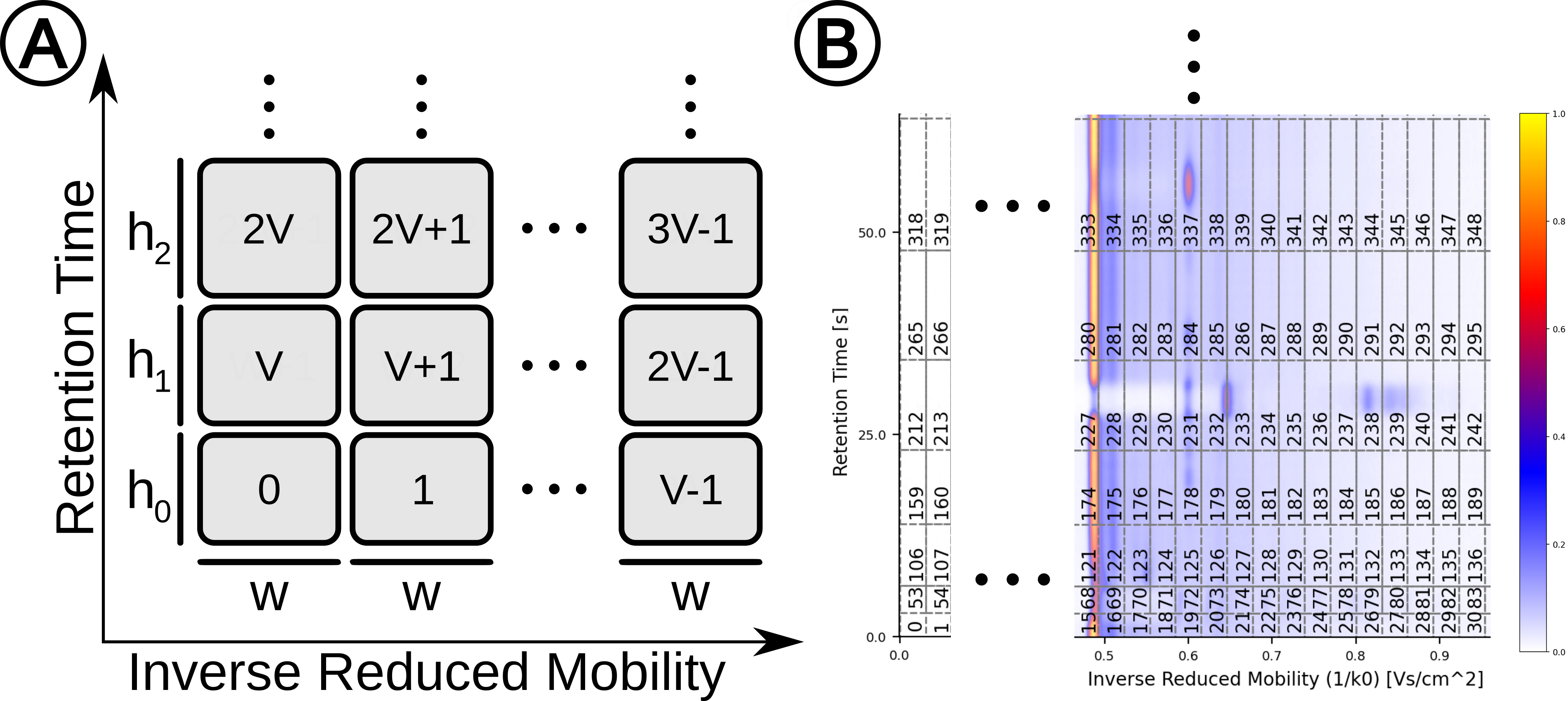

Supplement: Supplementary file 1 [file metabolites-10-00393-s001.zip › figure_S2.png]

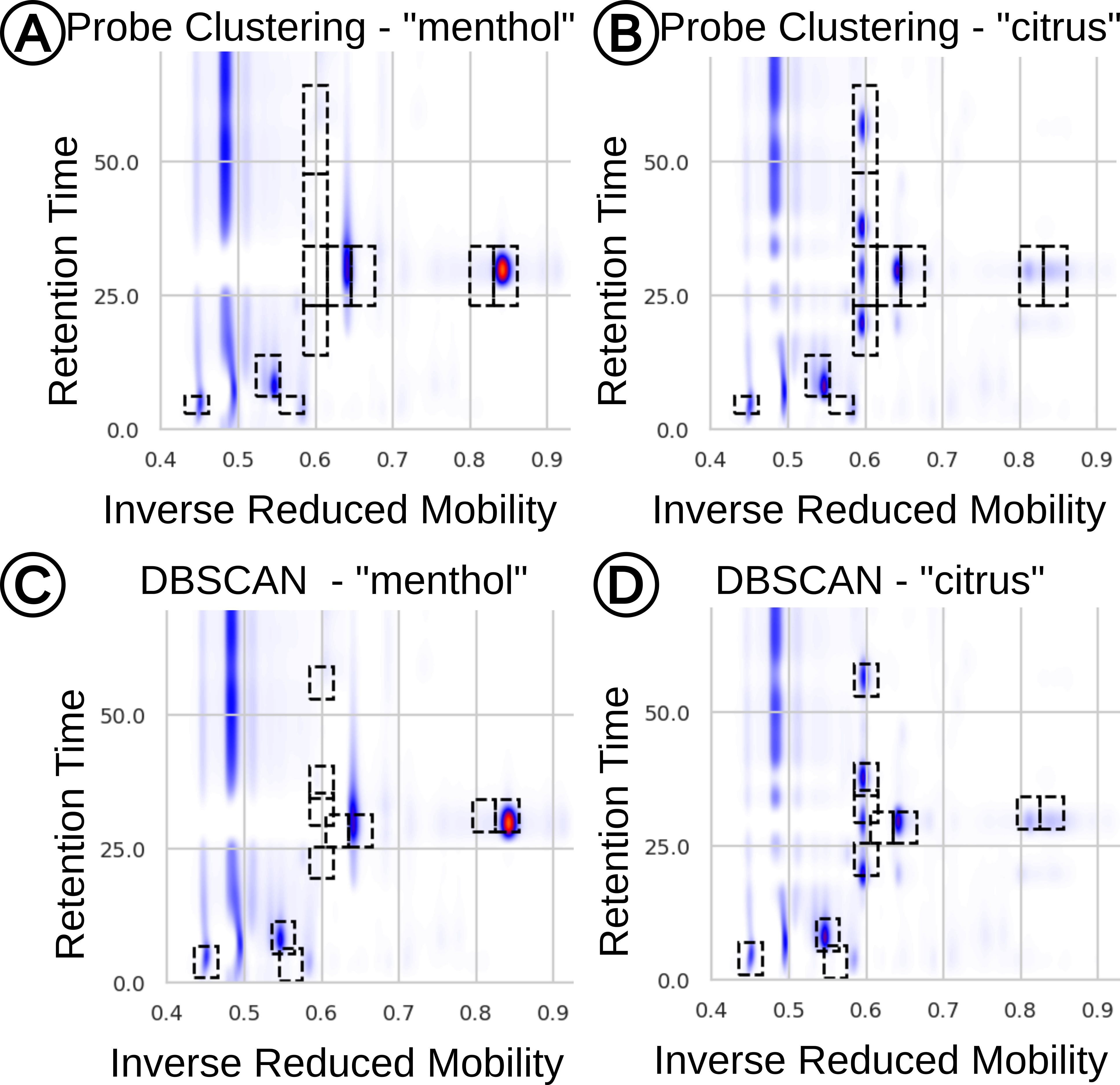

Supplement: Supplementary file 1 [file metabolites-10-00393-s001.zip › figure_S1.png]
